# Ovine Forestomach Matrix Graft Reduces Surgical Dehiscence in Fasciocutaneous Flap-Based Closure of Pilonidal Disease: A Comparative Study

**DOI:** 10.7759/cureus.96775

**Published:** 2025-11-13

**Authors:** Yosef Nasseri, Kimberly Oka, Kristina La, Rachel Ma, Christopher Frampton, Jessica Simon, Adam Young, Moshe Barnajian

**Affiliations:** 1 Colorectal Surgery, Cedars-Sinai Medical Center, Los Angeles, USA; 2 Department of Psychological Medicine, University of Otago, Christchurch, NZL; 3 Department of Medical Affairs, Aroa Biosurgery Limited, Mangere, NZL

**Keywords:** advancement flap, bioscaffold, fasciocutaneous flap, ovine forestomach matrix, pilonidal sinus disease

## Abstract

Background

Fasciocutaneous flap advancement is a newer alternative surgical approach in pilonidal sinus disease (PSD). This comparative study aimed to assess whether the use of an ovine forestomach matrix (OFM) graft implanted beneath a fasciocutaneous flap closure could minimize dehiscence complications in PSD.

Methods

PSD patients who received OFM and a fasciocutaneous flap were prospectively enrolled. Findings were compared to retrospective data from control patients who did not receive an OFM graft. Study outcomes were time to heal, incidence of dehiscence, and scar cosmesis. A within-study cost-utility analysis estimated costs and quality-adjusted life-years (QALYs).

Results

A total of 46 patients who received a flap advancement and OFM graft were compared to 48 retrospective control patients who were treated with flap advancement alone. No difference in median healing time was found in the OFM group (8.0 (IQR: 4.2, 15.8) weeks) compared to the control group (8.0 (IQR: 6.0, 15.2) weeks) (p=0.79). The incidence of dehiscence was significantly less in the OFM group (18%) versus the control group (46%) (p=0.005). The probability of dehiscence in the control group was 3.2 times greater compared to the OFM group. In a cost-utility analysis, the OFM group dominated the control group, with higher QALYs (0.24 vs. 0.23) and lower costs ($4,157 vs. $4,219).

Conclusions

The use of OFM graft with a fasciocutaneous flap resulted in a significantly lower incidence of dehiscence and potentially higher quality-of-life and cost savings, while maintaining a midline incision with favorable cosmesis.

## Introduction

Pilonidal sinus disease (PSD) is an acquired condition characterized by chronic inflammation and infection of the sacrococcygeal region resulting in pain and draining sinus tracts. With an incidence of 26 to 100 per 100,000 individuals in the United States, PSD represents a significant number of diagnoses per year [1]. Although PSD is a benign condition, disease progression and a high recurrence rate can result in chronic infection, polymicrobial abscesses, and multi-treatment modality failure [2]. For these reasons, numerous advances have been made to offer definitive treatment and reduce complications and recurrence. Conservative non-excisional intervention includes deroofing, endoscopic and laser treatment, crystallized phenol, and thrombin gelatin matrices as first-line treatments; however, in severe cases, PSD will fail conservative management and require surgery [3]. Once surgery becomes necessary, a lack of consensus on the optimal surgical procedure remains [2].

Excision with primary closure, which includes all flap-based procedures, is the current mainstay when surgery is elected. The Karydakis flap is a commonly employed flap technique that lateralizes the closure and scar off midline to a single side and has been reported to result in more favorable cosmesis, quicker healing times, and shorter return-to-activity [4,5]. Conversely, the Limberg flap scar crosses the midline and has been associated with less favorable cosmesis, but lower rates of recurrence and complications [2]. However, other meta-analyses suggest there is minimal difference between the two flap techniques [6]. Another technique is excision with midline closure, but it has been associated with a high rate of recurrence [7]. Combining the benefits of a flap technique and the cosmesis of a midline closure, a bilateral gluteal fasciocutaneous flap with midline closure is another approach [8]. Regardless of flap technique, complications including infection, seroma, dehiscence, and recurrence remain a major concern [2]. For example, wound dehiscence rates from various flap-based closures have been reported as high as ~10-36% [6,9,10]. The prolonged treatment course as a result of these complications adds to the overall burden of the disease.

One potential solution to the dehiscence rates observed in flap-based reconstruction is augmenting tissue healing with extracellular matrix (ECM) grafts. Insertion of the graft below the level of the advancement flap could potentially eliminate dead space and enhance the formation of well-vascularized tissue, thus increasing the probability of flap success. The elimination of dead space and rapid tissue formation could, in theory, reduce the rate of seroma formation, dehiscence, and recurrence [11]. Ovine forestomach matrix (OFM) is a mammalian-derived decellularized ECM that retains native structural architecture while maintaining signaling molecules supporting tissue regeneration [12,13]. OFM has previously demonstrated success in PSD reconstruction with OFM graft implanted below gluteal fasciocutaneous advancement flaps with minimal complications and favorable cosmesis [11]. Therefore, OFM implantation under a flap may be a viable option for patients who have previously failed conservative management and are particularly at risk of developing post-operative complications, thus favoring more positive short-term and long-term healing outcomes compared to other treatment options.

This comparative study sought to investigate the efficacy and cost-utility of using implanted OFM to augment a previously described bilateral gluteal fasciocutaneous advancement flap with suture tie-over and midline closure technique in PSD patients requiring surgical intervention [8]. The objective of the study was to evaluate whether implantation of an OFM graft could decrease the relatively high dehiscence rate associated with midline PSD reconstruction [5,7], while maintaining the favorable cosmetic outcome of a midline scar.

## Materials and methods

General

The comparative control group, who received gluteal fasciocutaneous advancement flap with midline closure alone, represents a previously published retrospective cohort from April 2014 to June 2020 [8]. Participants receiving the same advancement flap and implanted OFM graft were enrolled prospectively in an IRB-approved and HIPAA-compliant observational registry (NCT05243966) from March 2022 to April 2024. All surgeries were performed consecutively by two senior surgeons (YN, MB) at a single institution following the same surgical protocol. All prospective participants provided informed consent and had either previously failed conservative management or not been a candidate due to the extent of the disease. Inclusion and exclusion criteria are provided in Table 1. Primary endpoints included time to heal (weeks) and surgical site dehiscence. Complete healing was defined as 100% wound epithelialization and absence of drainage. Wound dehiscence was defined as any opening larger than the space between two sutures at the time of original wound closure. Descriptive statistics were computed using GraphPad Prism (v10.1.2, GraphPad Software, LLC, La Jolla, CA, USA). Kaplan-Meier survival analysis was conducted using SPSS Statistics version 28 (IBM Corp., Armonk, NY, USA). Continuous variables with normal distribution were presented as mean (SD); non-normal variables were reported as median (IQR). For categorical variables, p-values were determined using chi-square tests; for continuous variables, significance between treatment groups was determined using independent t-test or the nonparametric Mann-Whitney U-test. A two-tailed p-value <0.05 was taken to indicate statistical significance.

**Table 1:** Inclusion and Exclusion Criteria PSD: pilonidal sinus disease, OFM: ovine forestomach matrix.

Inclusion Criteria	Exclusion Criteria
Patient age ≥18 years of age	Received a surgical repair technique other than a fasciocutaneous advancement flap
Diagnosis of PSD	Patients with wounds with uncontrolled clinical infection (CDC Contamination Grade=4)
Received a fasciocutaneous advancement flap with or without OFM graft	Any medical condition or serious intercurrent illness that, in the opinion of the investigator, may make it undesirable for the patient to participate in the study
	Patient is currently participating or has participated in another clinical study within the past 30 days prior to enrollment
	Pregnant or lactating women
	Known allergy to sheep products

Surgical technique

**Figure 1:**
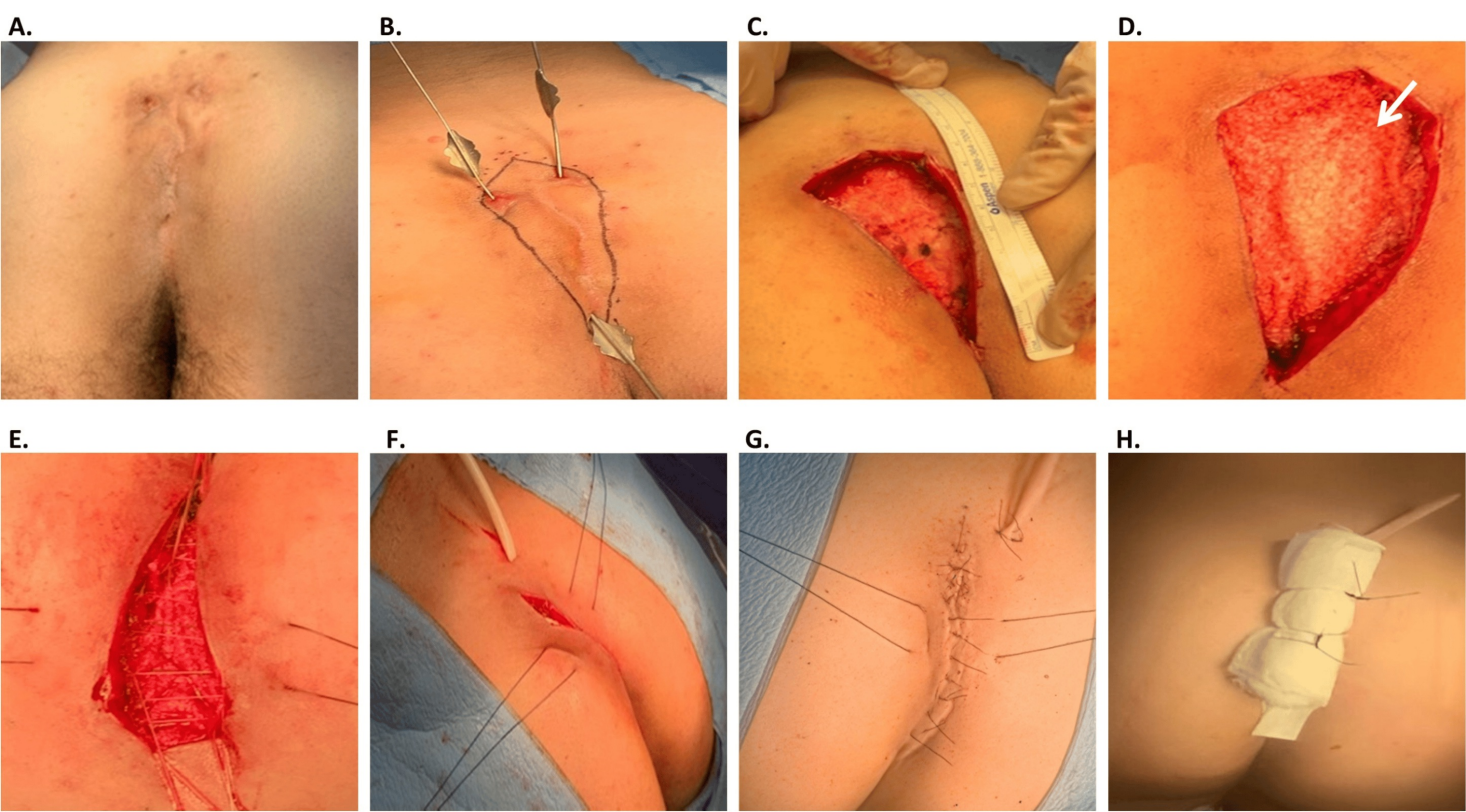
Surgical Procedure for Implanting Ovine Forestomach Matrix (OFM) Graft in Pilonidal Sinus Disease (PSD) Reconstruction with Midline Closure (A) Initial defect. (B) Identification of sinus tract and excision planning. (C) Wide excision of the PSD. (D) Placement of OFM graft into the defect site; arrow indicates the OFM graft. (E) Progressive tension sutures for the bilateral gluteal advancement flap. (F) Drain placement. (G) Midline closure. (H) Bolster dressing with suture tie-over technique.

All participants received a bilateral gluteal fasciocutaneous advancement flap with midline closure and suture tie-over as described in our prior study [8]. Briefly, the initial defect within the sacrococcygeal region was identified (Figure 1A), including any tracts or abscess cavities, and the precise borders were delineated (Figure 1B). A wide surgical excision was performed, extending down to the fascial level to ensure complete removal of all diseased tissue (Figure 1C). The defect was irrigated with normal saline. Bilateral fasciocutaneous flaps were bluntly created in a 360-degree fashion to facilitate a tension-free closure. For those patients receiving an OFM graft (Myriad Matrix^TM^ 3-layer, 10x10 cm; Aroa Biosurgery Limited, Mangere, New Zealand), the graft was rehydrated in sterile saline and placed at the bottom of the defect (Figure 1D). Notably, all patients received the same size of OFM graft following wide excision of the sinus to standardize the surgical technique across the study regardless of wound size. After the OFM graft application, two layers of 2-0 Vicryl® sutures were employed (Figure 1E). The first layer aimed at bringing the flaps together in a tension-free manner while simultaneously incorporating the OFM graft. Prior to tightening the second layer of sutures, a Jackson-Pratt® drain was selectively placed (Figure 1F). Two non-absorbable polydioxanone (1-0), full-thickness tie-over sutures were placed at the lower portion of the wound to manage tension without yet tying over (Figure 1F). Following the closure of the subcutaneous tissue, the skin was closed using horizontal mattress 3-0 Nylon® sutures along the midline incision (Figure 1G). A bolster dressing was applied and secured with the tie-over sutures (Figure 1H). Patients were discharged the same day. Postoperatively, patients were counseled to refrain from directly sitting on the site for 48-72 hours. Tramadol and ketorolac were given for pain management. Tie-over sutures and drain were removed in-office on post-operative day five and one to two weeks, respectively. Weekly follow-up was conducted until the wound was completely healed. Patients were monitored for up to 12 months for post-operative complications, recurrence, patient-reported pain score (Likert scale, 0-10), patient-reported scar satisfaction (Likert scale, 1-5), and observer cosmesis scores (modified Vancouver scar scale, VSS).

Cost-utility analysis

A cost-utility modeling analysis was conducted using Excel (Microsoft, Redmond, WA, USA), using published utilities. Procedure and post-operative care costs were estimated using Centers for Medicare and Medicaid Services published reimbursement rates. Cost-utility outputs included procedure, follow-up care, total costs, and quality-adjusted life years (QALYs) for each group along with differential costs and QALYs. An incremental cost-utility ratio (ICUR) was calculated as incremental costs divided by incremental QALYs.

## Results

General

There were 48 patients included in the retrospective control group and 46 patients were enrolled in the OFM group (Table 2), with no significant difference in demographic data between cohorts (p>0.05). There was no statistically significant difference in the median time to heal between the control group, 8.0 (IQR: 6.0, 15.2) weeks (n=48), and OFM group, 8.0 (IQR: 4.2, 15.8) weeks (n=44) (p=0.79) (Table 3). One superficial infection occurred in the OFM group (n=1/46, 2.3%) that resolved uneventfully with topical antibacterial. There were no instances of recurrence in the OFM group at last follow-up (n=0/44). Two patients (4%) were lost to follow-up in the OFM group, one prior to complete closure, and the other prior to a meaningful determination of wound dehiscence. The median follow-up time was significantly longer in the control group (145.5 (IQR: 105.3, 251.0) weeks) compared to the OFM group (15.5 (IQR: 13.0, 28.0) weeks) (Table 3).

**Table 2:** Patient Demographics For categorical variables, p-values were determined using chi-square tests; for continuous variables, significance between treatment groups was determined using independent t-test or the nonparametric Mann-Whitney U-tests. A two-tailed p-value <0.05 was taken to indicate statistical significance. Abbreviations: SD, standard deviation; BMI, body mass index; n, sample size; OFM, ovine forestomach matrix.

	Control Group	OFM Group	p
n	48	46	-
Age (years) (mean±SD)	28.5±9.5	27.3±7.8	0.523
BMI (mean±SD)	27.0±6.2	27.0±4.8	0.969
Gender			
Female, n (%)	9 (19%)	9 (20%)	0.920
Male, n (%)	39 (81%)	37 (80%)	
Smoker			
Never, n (%)	39 (81%)	40 (87%)	0.534
Current, n (%)	8 (17%)	6 (13%)	
Former, n (%)	1 (2%)	0 (0%)	

**Table 3:** Surgical Outcomes For categorical variables, p-values were determined using chi-square tests; for continuous variables, significance between treatment groups was determined using independent t-test or the nonparametric Mann-Whitney U-tests. A two-tailed p-value <0.05 was taken to indicate statistical significance. *Determined from Cox Proportional Hazards Regression. Abbreviations: SD, standard deviation; IQR, interquartile range; n, sample size; OFM, ovine forestomach matrix.

	Control Group	OFM Group	p
Maximum follow-up (weeks) median (IQR)	145.5 (105.3, 251.0)	15.5 (13.0, 28.0)	<0.0001
Time to heal (weeks) median (IQR) (n)	8.0 (6.0, 15.2) (48)	8.0 (4.2, 15.8) (44)	0.79
Dehiscence			
Yes, n (%)	22 (46%)	8 (18%)	0.005*
No, n (%)	26 (54%)	37 (82%)	
Time to dehiscence (weeks) median (IQR) (n)	2.5 (2.0, 4.0) (22)	4.0 (2.5, 4.8) (8)	0.091

Incidence of dehiscence 

**Figure 2:**
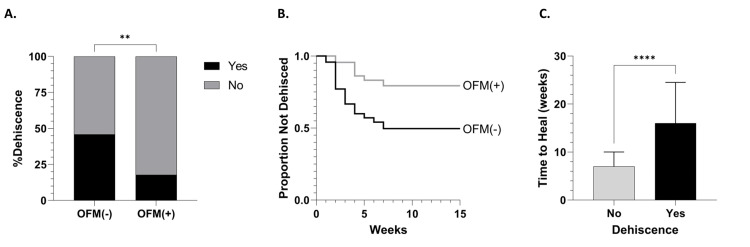
Dehiscence Analyses (A) Incidence of dehiscence in the control group and the OFM group. P-values were determined using chi-square tests, where differences were considered significant when p<0.05 (**, p<0.01). (B) Kaplan-Meier survival analysis of the proportion of PSD reconstructions that did not dehisce between the control group (black) and OFM group (grey). (C) Time to healing following PSD reconstructions where dehiscence occurred versus no dehiscence. Errors represent the IQR of the median.  P-values were determined using Mann-Whitney U-tests, where differences were considered significant when p<0.05 (****, p<0.0001). Abbreviations: IQR, interquartile range; OFM, ovine forestomach matrix.

The OFM group had a significantly lower incidence of dehiscence (18%, n=8/45) compared to the control group (46%, n=22/48) (p=0.005) (Table 3) (Figure 2A). A Kaplan-Meier curve showed the cumulative non-dehiscence for OFM compared to the control cohort (Figure 2B), demonstrating early and sustained separation between groups, with higher non-dehiscence probability in the OFM cohort. Cox proportional hazard analysis computed a hazards ratio of 3.23 (95%CI 1.43, 7.28, p=0.005). The median time to dehiscence was longer in the OFM group (4.0 (IQR: 2.5, 4.8) weeks) versus the control group (2.5 (IQR: 2.0, 4.0) weeks) and approached significance (p=0.091) (Table 3). Median time to complete healing was significantly longer for those reconstructions that did dehisce compared to those that did not (7.0 (IQR: 4.0, 10.0) versus 16.0 (IQR: 10.0, 24.5) weeks) (p<0.0001) (Figure 2C).

Patient-reported outcomes and scar cosmesis

For the OFM group, patient-reported pain was median 0 (IQR: 0, 1) (n=35) at last follow-up visit (Table 4). Patient-reported scar satisfaction was high, with a median score of 5 (IQR: 5, 5) (n=17) (Table 4). Observer reported scar score (VSS) was 100% normal vascularity, 95% supple, 86% flat scar height, and 95% normal pigmentation (Table 4).

**Table 4:** Observer and Patient Reported Scar Scores and Pain Score for the OFM Group Abbreviations: IQR, interquartile range; n, sample size; OFM, ovine forestomach matrix.

Observer Scar Assessment Score	
Scar Vascularity	
Normal, n (%)	22 (100%)
Pink, n (%)	0 (0%)
Red, n (%)	0 (0%)
Scar Pliability	
Supple, n (%)	21 (95%)
Yielding, n (%)	1 (5%)
Firm, n (%)	0 (0%)
Banding, n (%)	0 (0%)
Contracture, n (%)	0 (0%)
Scar Height	
Flat, n (%)	19 (86%)
<2 mm, n (%)	3 (14%)
2-5 mm, n (%)	0 (0%)
>5 mm, n (%)	0 (0%)
Scar Pigmentation	
Normal, n (%)	21 (95%)
Hypopigmentation, n (%)	0 (0%)
Hyperpigmentation, n (%)	1 (5%)
Patient Reported Pain (at last follow-up visit)	
Median (IQR) (n)	0 (0, 1) (35)
Patient Reported Scar Satisfaction (at last follow-up visit)	
Median (IQR) (n)	5 (5, 5) (17)

Cost-utility analysis

**Figure 3:**
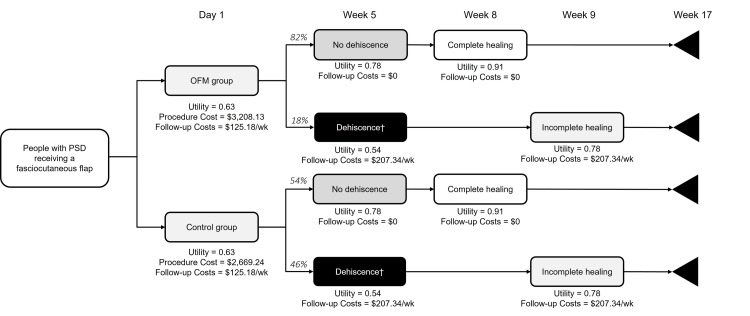
Cost-Utility Analysis Model Structure Decision time points are based on the time to dehiscence (~4.0 weeks, taken from Table 3) and median time to healing for patients that did (7.0 weeks) and did not dehisce (16.0 weeks). Follow-up costs consist of weekly office visits and/or cauterization (depending on dehiscence status). The utility decrement associated with dehiscence is only applied for four weeks, after which patients incur the quality-of-life benefit associated with incomplete healing. Abbreviations: PSD, pilonidal sinus disease; OFM, ovine forestomach matrix.

A cost-utility model considered four health states: post-surgery, incomplete healing without dehiscence, incomplete healing with dehiscence, and complete healing (Figure 3). Patients entered the model upon receiving a fasciocutaneous flap with or without an OFM graft. Patients then either did or did not dehisce at a frequency of 18% vs 82% for the OFM group, 46% vs 54% for the control group (Table 3). The model assumed dehiscence at week five, based on a median time to dehiscence of the OFM group of 4.0 weeks (Table 3). For those patients who did not dehiscence, complete wound healing was assumed to occur at week eight, based on time to healing where PSD reconstructions did not dehisce (Figure 2C). The model ended when all patients were assumed to be healed at week 17, based on time to healing where PSD reconstructions did not dehisce (Figure 2C).

Costs associated with the procedure, OFM graft, and follow-up care were included in the model (Table 5). The model assumed weekly follow-up outpatient office visits for the first four weeks. Additionally, it was assumed that for those patients who did dehisce, dehiscence would incur additional wound cautery for the first four weeks post-dehiscence, then weekly office-based wound care, until complete closure. Quality of life was assessed through literature-based health state utility indices (Table 5) [14-16]. The quality of life during normal wound healing was assumed at 0.78 [16]. The model assumed that the reduction in quality of life associated with wound dehiscence (utility, 0.54) was only experienced for four weeks. Upon complete healing, it was assumed that patients resumed the utility of the general US population (utility, 0.91), given that the OFM and control groups consisted of younger and generally healthy people [15].

**Table 5:** Cost-Utility Economic and Utility Inputs *Procedure costs inclusive of the cost of the OFM graft. Abbreviations: CPT, current procedural terminology; OFM, ovine forestomach matrix.

Economic Inputs ($USD) [17]	Unit Cost
Procedure Costs	
Excision of complicated pilonidal cyst and sinus, CPT 11772	$749.47
Bilateral fasciocutaneous gluteal flap, CPT 15734-59	$1,466.27
Complex wound closure measuring 8 cm^2^, CPT 13132	$453.50
Surgical preparation of recipient site for graft*, CPT 15002	$331.23
Implantation of biological implant*, CPT 15777	$207.66
Follow-up Care Costs	
Outpatient office visit, CPT 99214	$125.18
Chemical cauterization of granulation tissue, CPT 17250	$82.16
Utility Inputs	
Post-Surgery [16]	0.63
Incomplete healing	
Dehiscence [14]	0.54
No Dehiscence [16]	0.78
Complete Healing [15]	0.91

Over ~17 weeks, the OFM group was associated with a total per-patient cost of $4,157, consisting of $3,028 and $949 in procedure and follow-up care costs, respectively, and 0.24 QALYs (Table 6). The control group was associated with a total per-patient cost of $4,219, consisting of $2,669 and $1,550 in procedure and follow-up care costs, respectively, and 0.23 QALYs. Incrementally, the OFM group had $539 greater per-patient procedure costs, due to additional cost of the OFM graft. However, follow-up care was reduced by $601 compared to the control group, resulting in -$62 incremental per-patient total costs associated with the OFM group. The OFM group was also associated with 0.01 more QALYs (0.24 vs 0.23 QALYs) (Table 6).

**Table 6:** Per-Patient Cost-Utility Analysis Costs and QALYs for OFM and Control Groups *Because OFM was more effective and less costly than controls, reconstruction with OFM is considered ‘dominant’ from an economic modelling perspective. Abbreviations: ICUR, incremental cost-utility ratio; NA, not applicable; QALYs, quality-adjusted life-years; OFM, ovine forestomach matrix.

	OFM group	Control group	Difference
Total Cost ($USD)	$4,157	$4,219	-$62
Procedure Costs	$3,208	$2,669	$539
Follow-up Care Costs	$949	$1,550	-$601
QALYS	0.24	0.23	0.01
ICUR	NA (OFM dominates control)*		

## Discussion

This comparative study evaluated outcomes following a fasciocutaneous flap and midline closure with or without an implanted OFM graft for PSD reconstruction. All patients had previously failed conservative or surgical treatments. As expected, the follow-up time was significantly longer in the retrospective control group versus the prospective group that received an OFM graft (~145 weeks versus ~15 weeks, respectively, Table 3). This was simply due to the time elapsed since the onset of data collection in the retrospective cohort. Surgery across all patients was performed by the same two senior surgeons, using the same operative protocol, perioperative care, and post-operative management, aside from the addition of the OFM graft to patients in the prospective group. The retrospective control group was treated consecutively prior to prospectively enrolling patients in the OFM group. This study design reduced the potential for variability due to any surgical learning curve or evolving surgical technique between both groups as both surgeons had established and standardized their bilateral fasciocutaneous flap procedure prior to control group enrollment.

Median time to heal was similar between groups (Table 3), where healing was defined as complete epithelialization and no drainage. Only one superficial infection was reported in the OFM group, that resolved without additional surgical intervention. Although the rate of infection was not reported in the retrospective cohort, this low rate of infection falls at, or below, what is typically reported. For example, a recent meta-analysis comparing Karydakis and Limberg flaps reported variable infection rates from 2-24% [6].

The incidence of dehiscence and the probability of dehiscence were ~2.5x greater (Table 3) (Figure 2A) and 3.2x greater in the control group relative to those patients that received an OFM graft. These findings suggest that the addition of OFM graft offered a substantially decreased risk of wound dehiscence. Similar to the stringent definition of time to heal, dehiscence was defined as a breakdown equal to or greater than the distance of two sutures at the time of initial closure. This strict definition of dehiscence is likely one reason the 18% incidence of dehiscence in the OFM group is relatively high compared to published studies [4,7,18,19], many of which defined dehiscence more loosely, requiring a larger wound separation or the need for reintervention. By contrast, our conservative definition captured any wound separations greater than two sutures, likely increasing not only the reported incidence but also improving detection sensitivity.

In this study, a bilateral gluteal flap with midline closure was selected for favorable cosmesis. Unlike offline or midline crossing techniques, a midline closure scar follows the natural anatomic lines of the gluteal cleft. Cosmetic results are a major source of concern for patients, but cosmetic satisfaction remains a major challenge in PSD. For example, Alkurt et al. found that ~22% of patients were not satisfied with the outcome of a Limberg flap [4]. A study comparing midline closure with a Limberg flap reported a cosmetic score significantly lower in the Limberg group compared to the midline closure group (p<0.0001) [7]. In the current study, patient-reported scar satisfaction score in the OFM group was a median of 5 (IQR: 5, 5), indicating patients were highly satisfied with the cosmetic outcome and reinforcing cosmetic favorability of midline closure (Table 4). These data suggest that OFM graft used with a midline closure resulted in favorable scar cosmesis in the opinion of both the patient and the surgeon.

A cost-utility analysis compared the economic costs and quality-of-life benefits associated with patients in the OFM and control groups. On a per-patient basis, the OFM group was associated with $62 in cost savings and 0.01 in increased QALYs (~4% increase). By decreasing the rate of dehiscence, OFM may lead to cost savings through the reduced need for follow-up care and improved quality of life for patients. Follow-up wound care is not only a financial burden but also consumes healthcare resources. When estimating any intervention’s cost-utility, four scenarios are possible: (1) less costly and less effective, (2) more costly and less effective (i.e., dominated), (3) more costly and more effective, or (4) less costly and more effective (i.e., dominant) (Figure 4). Incremental costs and QALYs are plotted on the cost-utility plane (Figure 4). In the case of an intervention being more costly and more effective, an ICUR quantifies the trade-off between costs and health gains and is compared to national estimates of a country’s willingness-to-pay for an additional QALY gained (i.e., $100,000 to $150,000 in the US) to determine an intervention’s “value-for-money”. Given that OFM was more effective yet less costly than controls, OFM was considered ‘dominant’ and thus an ICUR was not needed.

There is a growing body of evidence to support the addition of OFM graft as an implant under tissue flaps, including PSD [11], hidradenitis suppurativa [20], pressure injuries [21,22], lower extremity reconstruction [23,24], and traumatic defects [25]. The rationale is to use the graft to fill surgical dead space and therefore reduce the risk of post-operative complications. This hypothesis is supported by controlled in vivo studies that have previously shown that the addition of ECM graft to surgical dead space significantly reduces post-operative complications [26,27]. In addition to eliminating surgical dead space, it is posited that another mechanism of action of the implanted OFM graft could be the generation of vascularized tissue at the level of the flap/graft interface. OFM supports rapid cellular infiltration and has demonstrated a known ability to stimulate angiogenesis leading to increased vascularity and tissue remodeling [28]. Additionally, OFM is known to contain a large number of different cytokines including VEGF, PDGF, KGF, and BMPs, that may be contributing to improved flap integration [12]. While the current study focused on the use of OFM in reducing dehiscence when placed under a gluteal fasciocutaneous flap, the positive outcomes herein suggest a potential broader utility for OFM in other reconstructive closures. For example, flap-based reconstructions and closures, including total or hemi-arthroplasty hip surgery, abdominoplasty, spine surgery, lipoma/sarcoma excision, and many others present their own challenges with respect to post-operative seroma, hematoma, dehiscence and tissue necrosis. As we have shown in the current study targeting PSD, implanted OFM graft may be a cost-effective means of reducing post-operative complications in these procedures as well.

Limitations

While recurrence remains a significant long-term outcome in pilonidal disease, the scope of this analysis was focused specifically on short-term outcomes including wound dehiscence and patient satisfaction. Additionally, the single-center nature of the analysis constrains generalizability, as it did not consider variability in surgical technique between different institutions. Moreover, the use of a retrospective control group may have also introduced confounding despite a consistent and established surgical technique across all patients. By utilizing a retrospective control group the overall power of the study was limited as additional patients could not be added to the control cohort. While statistical significance of the primary endpoint was ultimately achieved, the results should be interpreted cautiously due to potential overestimation of the effect size. Furthermore, the cost-utility analysis does not consider a societal perspective, such as ability to pay and impact on return to work due to follow-up care. Reductions in the rate of dehiscence may intuitively reduce travel costs and productivity losses, thereby increasing societal cost savings.

## Conclusions

Implanted OFM graft with a fasciocutaneous advancement flap and midline closure significantly reduced the incidence of wound dehiscence compared to flap closure alone, offering both clinical and economic benefits in the surgical management of pilonidal sinus disease. By decreasing the risk of wound breakdown, OFM may lessen the burden of prolonged wound care, reduce the need for follow-up interventions, and improve patient quality of life. The cost-utility analysis further supported the OFM technique, demonstrating lower overall costs and incremental quality-of-life gains relative to controls.

While this study focused on pilonidal sinus disease, the findings suggest that OFM grafting should be explored in other flap-based reconstructions at risk of postoperative complications. The ability to fill dead space, promote angiogenesis, and support tissue integration may position OFM as a promising adjunct for reducing surgical complications and supporting patient satisfaction and should be further researched.
